# Highly Reflective Silver-Enhanced Coating with High Adhesion and Sulfurization Resistance for Telescopes

**DOI:** 10.3390/nano12071054

**Published:** 2022-03-23

**Authors:** Hsing-Yu Wu, Shao-Rong Huang, Chih-Hsuan Shih, Li-Jen Hsiao, Hong-Wei Chen, Ming-Chung Cheng, Jin-Cherng Hsu

**Affiliations:** 1 System Manufacturing Center, National Chung-Shan Institute of Science and Technology, New Taipei City 237209, Taiwan; andy810301@gmail.com (H.-Y.W.); hslijen@gmail.com (L.-J.H.); 2 Center for Astronomical Physics and Engineering, National Central University, Taipei 320317, Taiwan; oz1111zonew@gmail.com; 3 Department of Electro-Optical Engineering, National Taipei University of Technology, Taipei 10608, Taiwan; 4 Department of Physics, Fu-Jen Catholic University, New Taipei City 242062, Taiwan; zxc959647833@gmail.com (C.-H.S.); zebra45678988@gmail.com (H.-W.C.); 5 Donell Optronics Co., Ltd., 6F.-7, No. 13, Shangshi N. 2nd Ln., Sec. 2, Xitun Rd., Xitun Dist., Taichung City 40749, Taiwan; tommy@donelloptro.com; 6 Graduate Institute of Applied Science and Engineering, Fu Jen Catholic University, New Taipei City 242062, Taiwan

**Keywords:** silver mirror, Ag-enhanced coating, telescope, sulfurization resistance

## Abstract

Highly reflective metal coatings are essential for manufacturing reflecting telescope mirrors to achieve the highest reflectivity with broad spectral bandwidth. Among metallic materials, enhanced silver-based coatings can provide higher reflectivity in the 400–500 nm spectral range to better performance from visible to near IR. Moreover, over-coating a dielectric protective layer on the mirror’s front side attains additional hardness and oxidation stability. In this paper, we study a combination of thermal and electron beam evaporation as a technology to form protected enhanced high reflective Ag coatings. A newly designed multiplayer film can pass ASTM 5B adhesive performance testing and give sulfurization inhibition. The average specular reflectivity for the enhancement coating is about 98% in wavelengths across the spectral range from 400–1000 nm. This innovation has been demonstrated on a Newtonian type telescope, with storage in an ambiance humidity H = 60–85%, and temperature T = 10–35 °C, for more than six months without degradation in coating performance.

## 1. Introduction

Telescopes are deployed in space, on the ground, and in marine environments to increase our capacity to observe objects from a long distance. The next generation of innovative and mass production projects concern the development of high reflectivity across the light spectrum from the near-infrared to the visible range [1,2,3,4]. The devised technique has great potential to be used in such a system. The fundamental importance of these telescopes is their primary mirrors as they decide system sensitivity, which is directly proportional to their size [5,6,7,8]. In most applications, the mass and mechanics can usually constrain the size of the primary mirrors, and metallic mirror coatings can improve that.

Metallic coated mirrors have been widely used in the reflective imaging system to minimize loss while reflecting light sources from a dim observing space. To reduce system loss, the use of a high-reflective dielectric coating to collect more light is the current method of increasing reflectivity. So far, aluminum is the most cost-effective coating material for all telescopes, with an extensive reflectivity range covering ultra-violet to mid-infrared [9,10,11]. However, lower averaging reflectivity above 400 nm wavelengths and dropping reflectivity around 850 nm occurs due to the interband transitions of Al stack coatings [12,13].

Silver coatings may be a suitable substitution for the Al coating. Unfortunately, our environments have the molecules of O, O^−^, H_2_O_2_, SO_2_, and Cl^−^ in the air resulting in AgCl, AgSO_4_, AgNO_3_, or Ag_2_S to degrade the film qualities [14,15,16,17]. Developing stacks of thin films is the current method used to achieve the required high-quality Ag coatings. For example, adding adhesion layers between the mirror substrates and the metallic Ag layer can achieve a robust coating. The high/low-index stack multilayer can enhance the silver reflectivity in the blue spectrum and achieve overall high reflectivity in the applied spectral region. In addition, the protective layers can avoid the Ag coatings from the presence of sulfur in the ambient.

This paper investigates the innovative enhanced dielectric Ag coatings designed, simulated, and deposited by thermal and electron beam evaporation technology. High reflectivity in the 400–500 nm spectral range and up to 98% averaging reflective rate in the 400–1000 nm is achievable and highly consistent, as demonstrated by the simulation results. In addition, adhesive and sulfurization performance testing to assess the stability of the Ag coating is conducted and shown in a Newtonian telescope for more than six months. The results show that the new visible-near IR enhanced and adhesion and sulfurization stability silver coating can prevent corrosions and oxidation for mirror assembling, transportation, and storage under various environmental conditions for the proceeding marine, ground, and space projects.

## 2. Materials and Methods

### 2.1. Experimental Setup

**Figure 1:**
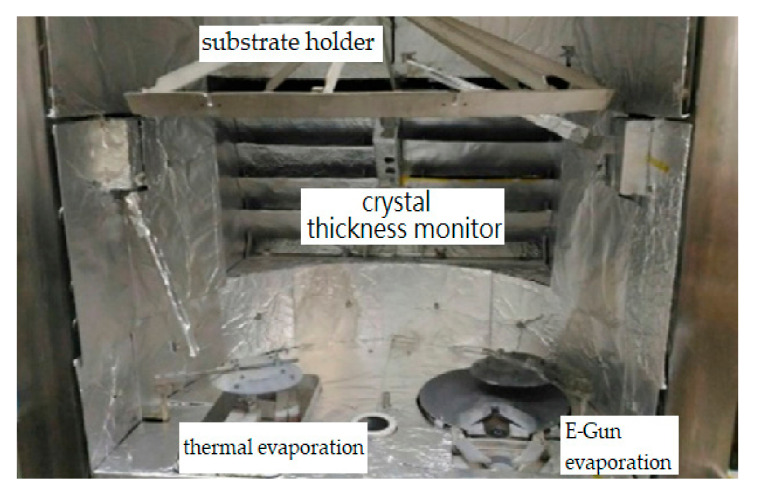
Combination of thermal and electron beam evaporation in the coating system.

In the discussion, the vacuum coating system is a 90 cm in diameter box coater (SGC-120SA-IAD, Showa Shinku Co., Yokohama, Japan) equipped with a 10 kW electron beam gun and an end-Hall ion source (Mark II) made by Veeco Ion Tech. Inc. (Plainview, NY, USA), as shown in Figure 1. The study used B270 glass in 25 mm diameter for testing, a primary mirror in 150 mm diameter, and the secondary mirror in 50 mm diameter of a telescope as substrates. First, we lightly polished the substrates with wet cotton moistened with CeO_2_ powder, and then ultrasonically cleaned it for 20 min. Next, the substrates, which were then blown with clean nitrogen gas, were settled on the rotating substrate holder approximately 60 cm above the deposition source. Before the deposition, it is essential to evacuate the vacuum chamber to a base pressure of 5 × 10^−4^ Pa. After that, the ion beam cleaning process for the substrates was performed about 35 min by a grid-less end Hall ion source Mark II (made by Veeco, Plainview, NY, USA) with Ar working gas. A combination of thermal evaporation for metal and electron beam evaporation for oxide material is the proposed technique for the silver coatings. Deposition materials with ion-assisted deposition (IAD) treatment can be physically heated and then transformed to vaporize evaporation materials to deposit the vaporized materials onto a substrate to form Ag multilayer films. Every film was fabricated by a different method, as shown in Table 1.

### 2.2. Protected, Enhanced Silver Coating Design

The design and how they simulate the reflectivity of Ag coatings were evaluated before a series of experiments to achieve consistency between modeling and the experimental results by examining the optical refraction indices of the as-deposited materials. First, a VASE ellipsometer (made by J. A. Woollam Co., Inc., Lincoln, NE, USA) measured the SiO_2_ and TiO_2_ layers. Their optical indices were 2.443 and 1.468 at 380-nm wavelength, respectively. Finally, The thicknesses of every layer were then designed and assisted by an optical thin film software (The Essential Macleod, vol. 9.7.0., Thin Film Center Inc., Tucson, AZ, USA) to achieve high reflectivity silver mirrors. Table 1 lists the deposition materials and their parameters of each layer for the Ag coatings.

**Figure 2:**
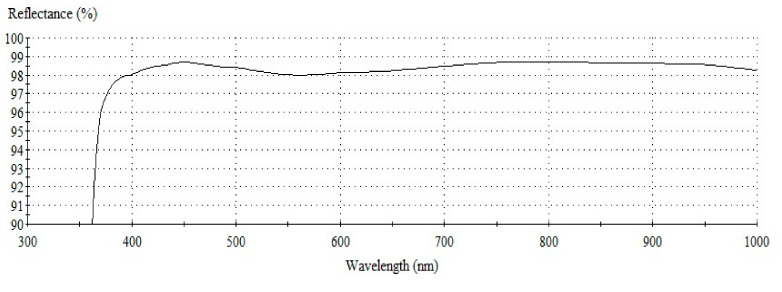
Design spectrum for high reflectivity silver mirrors.

Figure 2 shows the reflectivity of the simulation-based on Table 1 on a B270 substrate. The result indicates that the mirror achieves the minimum requirements over the entire wavelength range from visible to near IR. Furthermore, the reflectivity in the 450–1000 nm region is better than 98%, including the lower reflection region from 400–500 nm, which is much better than a pure silver mirror. Hence, the full coating stack Substrate/SiO_2_/Al_2_O_3_/Ag/Al_2_O_3_/SiO_2_/TiO_2_/SiO_2_/TiO_2_ was determined as the developed method to deposit a durable Ag-enhanced coating on all the mirror substrates. The following step is to determine the three different thicknesses (86 nm, 130 nm, and 200 nm) of the third Ag layers based on the environmental tests.

### 2.3. Sulfurization Test of the Ag-Coated Samples

**Figure 3:**
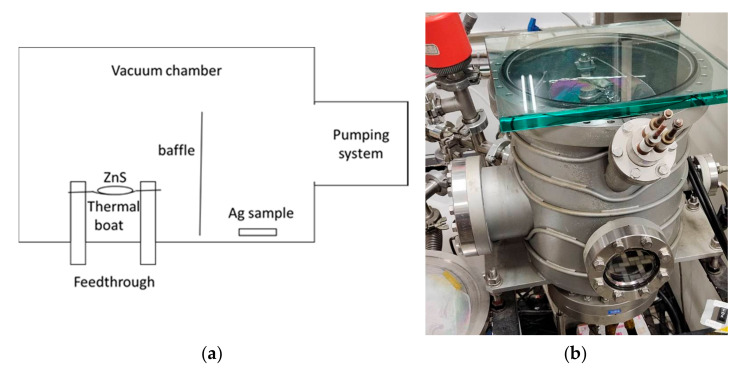
(**a**) schematic diagram of sulfurization chamber, (**b**) photo of the homemade sulfurization chamber.

As shown in Figure 3, a vacuum chamber with a diameter of 24 cm and a height of 28 cm was used to assess the Ag coatings for the discriminating environmental tests. Samples of 86 nm, 130 nm, and 200 nm, respectively, were deposited on the multilayers by the optical coating system shown in Figure 1, and then placed on the other side of the baffle as shown in Figure 3. First, we loaded a tungsten boat with zinc sulfide (ZnS) of 0.3 g. Next, the sulfurization testing chamber was evacuated to a base pressure of 5 × 10^−3^ Pa and turned off the pump valve to keep the chamber vacuum condition at room temperature. Following that, we heated the tungsten boat to evaporate the whole zinc sulfide. A stable quantity of sulfide gas decomposed from the evaporation in the chamber to make contact with the Ag samples for 10 min.

### 2.4. Adhesion Test of the Ag-Enhanced Multilayer

Adhesion can be one of the most critical factors to assess the applications of coatings. We cut Ag-enhanced multilayer on the samples by 1 mm × 1 mm. The pull-off tests using a 3M 600 transparent tape based on the ASTM standard were used to examine the Ag coating method to a parabolic mirror for a Newtonian telescope.

### 2.5. Reflection Measurement of the Two Telescope Systems

We measured the reflection of the telescope optical system at the exit pupil by a spectrometer FLAME-S-XR1-ES (made by Ocean Optics Inc., Orlando, FL, USA), which has a linear silicon CCD array in the range of 200 nm–1.025 μm wavelength and 25-µm entrance slit, by using optical fiber and a collimator lens to detect a measured light. The detected object, combined with four sheets of A4 white paper, was located 40 m away at about five o’clock in the afternoon on a cloudy day.

### 2.6. Surface Roughness of Ag-Enhanced Telescope Mirror

The surface roughnesses of the two primary mirrors of the Newtonian telescope were measured by a white-light interferometric microscope Chroma 7502 (made by Chroma ATE Inc., New Taipei City, Taiwan).

## 3. Results, Analysis, and Applications

### 3.1. The Optical Thin Film Design of the Ag-Enhanced Multilayer

**Figure 4:**
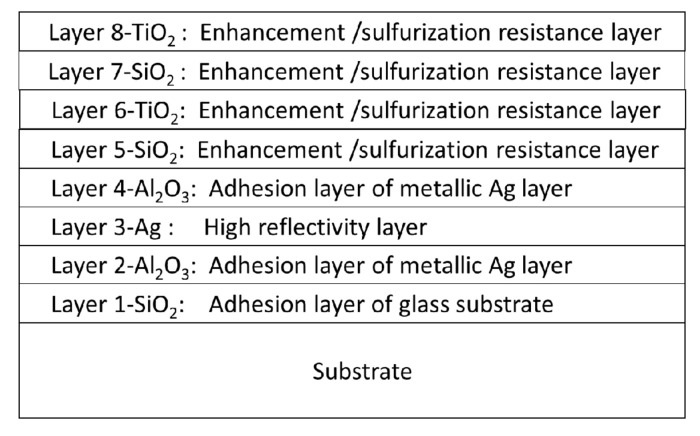
Schematic sketch of the functions at every layer in the Ag enhanced multilayer.

Figure 4 shows a schematic sketch of the functions at every layer in the Ag enhanced multilayer. Layer 1 and 2 are designed as buffer layers for binding glass substrate and the silver layer as the thin layer assists the adhesion of the silver layer in increasing the overall adhesion. The following Al_2_O_3_ is also a buffer layer between the Ag layer and the stack of layers 5 to 8, SiO_2_/TiO_2_/SiO_2_/TiO_2_.

The thicknesses of the designed Layer 3 are 86, 130, and 200 nm. Layers 1 and 2 do not affect the multilayer reflectivity as they are below Layer 3, almost opaque for their thicknesses. The designed optical admittances (*N* = *n*-i*k*, *n* is an index, *k* is extinction coefficient), reflectance, and phase of Ag layers deposited on Layer 2 with 86, 130, and 200 nm are almost the same, as shown in Table 2. Therefore, the same Layers 5 to 8 were designed for enhancement with the optimization program of the software, as shown in Table 1.

**Figure 5:**
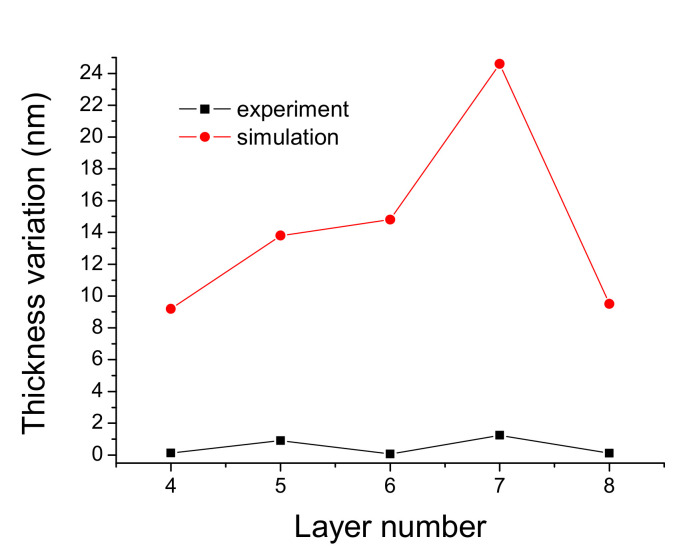
Comparison of the thickness variation in simulated layers 4–8 by reducing 0.5% reflectivity to the thickness variation in the experimental layers controlled by an optical monitor.

Figure 5 shows the thickness variations simulated by reducing a 0.5% reflectivity for Layers 4 to 8 compared with the thickness variations produced during deposition. The experimental thickness variation in each layer is the standard deviation value, which is the statistic of the optical monitoring intensities at the end of several coats, normalized by the maximum intensity variations in the deposition, and then approximately multiplied by the thickness of the quarter wavelength.

### 3.2. The Reflectivity of the Ag-Coating Affected by Sulfurization Test

Compared to the commercial ANSI/EIA-977 test [18] for the sulfurization treatment, the test duration is 480 h at 60 °C or 750 h at 105 °C by corrosive sulfur gas. Our system can quickly measure the sulfurization resistance of the sample in an hour. It is easily used and combined with our laboratory. A low humidity level is assumed to be present in the vacuum chamber; the sulfur gas decomposed from the same quantity of zinc sulfide can stably react with the test sample at room temperature.

**Figure 6:**
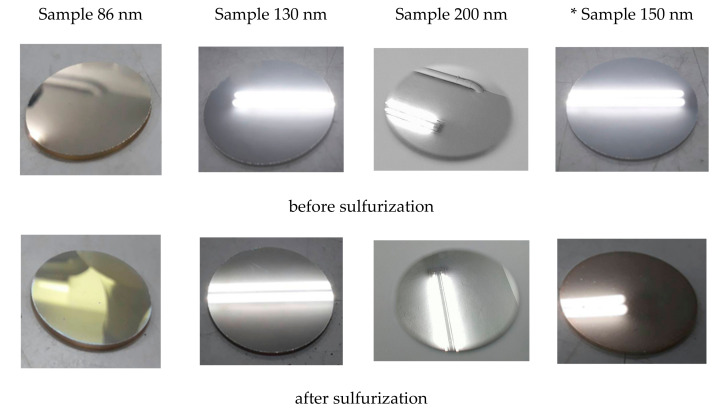
Before sulfurization, the image above shows the third layer of three samples deposited at 86 nm, 130 nm, and 200 nm thickness. * Sample 150 nm is a 150 nm thick silver film deposited on a B270 substrate. The figure below shows all the samples treated by the sulfurization process. The reflected image in the photo is the lamp above our laboratory.

The different samples were fabricated based on the technique reported in Section 2.1. Figure 6 lists the results of the sulfurization test, which were affected by the Ag-coated thickness of 86 nm, 130 nm, and 200 nm. In addition, we prepared another pure Ag layer sample with 150 nm thickness for further comparison.

**Figure 7:**
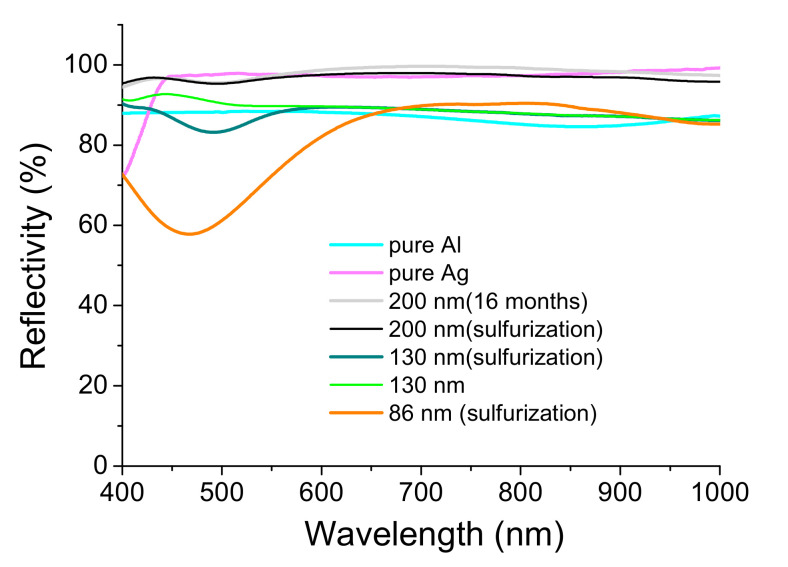
The reflectivity of sulfurized or untreated Ag-coated samples.

Figure 7 summarizes the reflectivity of the coated samples. The pure Ag mirror of Sample 150 nm presents the low reflectivity at the wavelength less than 450 nm. The pure Al mirror of the sample deposited by 150 nm thickness shows the low reflectivity in the spectrum. The Ag-enhancement coating can improve their defects. However, Sample 86 nm has a very low reflectivity of 480 nm wavelength after the sulfurization process. Sample 130 nm has an average reflectivity of about 90%. Its reflectivity also decreases from 470 nm to 520 nm and the lowest 82% at ~500 nm wavelength. Sample 200 nm has an average reflectance of approximately 97% after sulfurization treatment and humidity test greater than 70% at room temperature for 16 weeks.

### 3.3. Adhesion Test of the Ag-Enhanced Multilayer

A film cutter vertically and parallelly cut the enhanced Ag multilayer of the samples into multiple grids of 1 mm × 1 mm squares. After that, a 3 M 600 transparent tape was attached to the samples, pressed with about 60 N weight for 10 min, and then the tape was instantly torn off. Peeling was detected neither on the tape nor the sample’s edges between the incision and grid. Therefore, the adhesion was assessed to 5B.

### 3.4. Optical Reflectivity Comparison of the Ag-Enhanced and Pure Al Coatings

**Figure 8:**
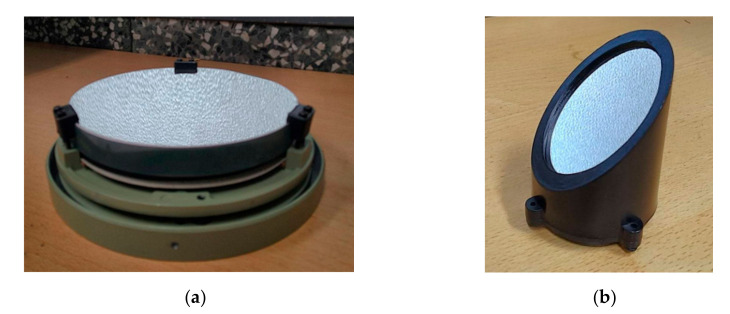
(**a**) Ag-enhanced primary mirror of the telescope, approximately 15 cm in diameter; (**b**) Ag-enhanced secondary mirror of the telescope, with a diameter of 5 cm.

To date, the samples for 16-month storage in 80% humidity have only an increase in some pinholes, which has a negligible effect on optical performance. However, the blue end in the 400–500 nm region does not degrade in reflectivity. In addition, Figure 8 shows the 150-mm aspherical primary mirror and secondary mirror. Both mirrors are highly reflective and pinhole-free when stored at 50% humidity for six months.

**Figure 9:**
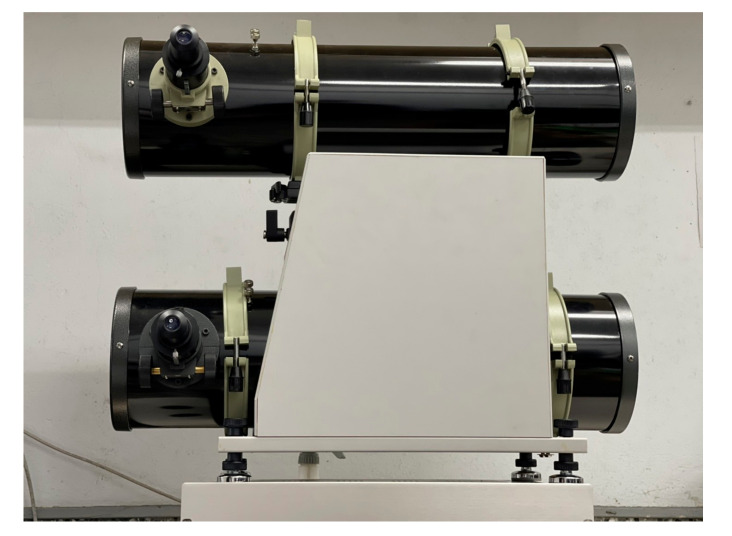
Double Newtonian telescope system: The primary and secondary mirrors of the upper telescope settled by Ag-enhanced mirrors and those of the lower fixed by Al-coated mirrors.

The pure Al mirror is usually used as a reflective mirror in the UV region. However, the average reflection is about 88% in the UV-visible region, as shown in Figure 7. The homemade double telescope system is shown in Figure 9. The primary and secondary mirrors deposited with silver-enhanced multilayers are placed in the upper telescope, while those deposited with pure aluminum films are in the lower telescope. The surface roughnesses (Ra) of the two primary mirrors in the 293 × 293 µm area are 1.18 ± 0.2 and 2.63 ± 0.2 nm, measured by the white-light interferometric microscope, respectively.

**Figure 10:**
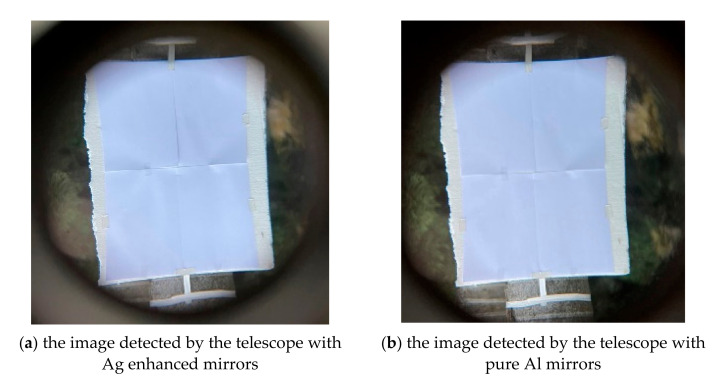
A prominent white object, combined with four sheets of A4 white paper, was hung next to a coconut tree and placed about 40 m away. Photo (**a**) taken with the Ag-enhanced system, and photo (**b**) taken with the pure Al-coated system.

Figure 9 shows the double Newton telescope system. The upper telescope uses the Ag-enhanced primary and secondary mirrors shown in Figure 8, and the lower one uses general aluminized primary and secondary mirrors. The upper telescope has a fine-tuning device and can adjust the telescope position and shoot the same object detected by the lower telescope for a long distance. In the upper Ag-enhanced coated Newtonian telescope system, the light intensity is reduced by about 3.6% as the two Ag-enhanced mirrors reflect the image with an average reflectivity of ~98%, shown in Figure 7. In the lower aluminized telescope system, the light intensity is reduced by about 22.6% as the imaging is reflected by two Al-coated mirrors with an average reflectivity of 88% shown in Figure 7. The Ag-enhanced system theoretically produces 19% greater intensity than the Al-deposited system. As such, and for further confirmation, the camera took these two pictures shown in Figure 10a,b compared through the double Newtonian telescope systems. A prominent white object was placed outdoors, approximately 40 m away. It is necessary to adjust the two images to be the same, as the comparative spectral measurements depend on the same two images.

**Figure 11:**
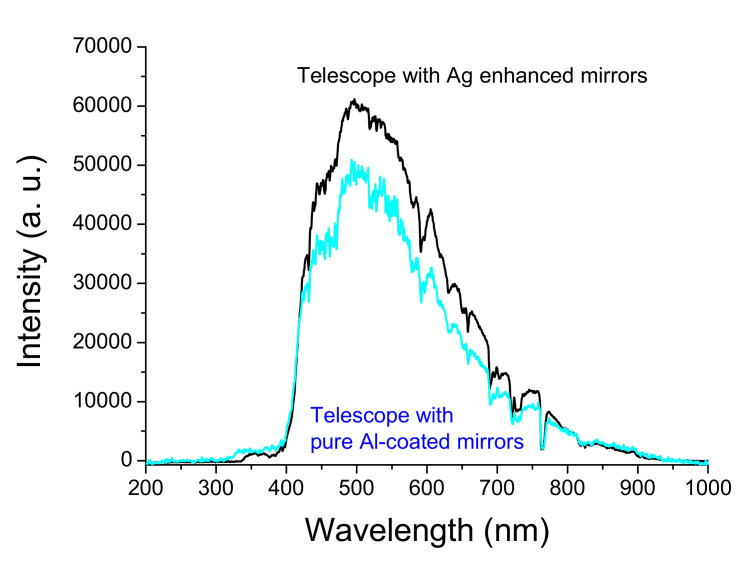
Spectra of two exit pupils of the double telescopes.

Figure 11 shows the exit pupil spectra of two Newtonian telescopes. The two curves were baselined at 200-nm and 1000-nm wavelengths. The spectral intensity of the telescope with two silver-enhanced multilayer mirrors, the intensity of 60,000 at 500-nm wavelength, is higher than the intensity of 48,743 for the telescope with pure Al-deposited mirrors. The intensity difference of 11,257 is about 19% normalized by the 60,000 value of the silver-enhanced system. The UV-region intensity of the Ag-enhanced system is somewhat less than that of the pure Al-deposited system due to the reflection of a pure Ag-deposited mirror in the region being relatively less than that of a pure Al-deposited mirror. However, at wavelengths greater than about 425 nm to 800 nm, the intensity of the Ag-enhanced system is greater than the Al-coated system.

## 4. Discussion

Table 1 shows the quick deposition rate of metallic silver significant under a low pressure of 5.5 × 10^−4^ Pa without IAD is beneficial to the excellent quality of metallic silver. Contrarily, the rate of TiO_2_ layers remains low to stoichiometry during the un-heating deposition [19,20]. The silver may be oxidized at a high substrate temperature larger than 200 °C and decreases the reflectance. Therefore, we kept the substrate temperature under 115 °C during the deposition, as shown in Table 1. Therefore, the optical indices of TiO_2_ and SiO_2_, lower than those deposited at 250 °C, are 2.443 and 1.447 at design 380-nm wavelength, respectively, in the IAD process with an ion beam voltage (V_b_) of 119.4 V and ion beam current (I_b_) of 2 to 3 A.

Figure 5 shows that the simulated variations are larger than the experimental variation in the optical monitoring uncertain values. The Ag-enhancement deposition is easier for fabrication than the optical bandpass filter [21,22] due to the considerable tolerance of the optical monitoring intensity for each layer deposition. However, Xu et al. have studied the adherence difference between Ag and MgF_2_, Al_2_O_3_, SiO_2_. They found SiO_2_ cannot be used as a protective coating due to its weak adherence and humidity for the metallic Ag layer [23,24]. In Figure 4, Layer 2 and 4 are designed as buffer layers on the silver layer for binding glass substrate and the enhancement stack. However, the reflectivity in the region of 400 to 500 nm decreases with an increase in the thickness of the Al_2_O_3_ layer. The multilayer adhesion will reduce if the Ag-enhanced multilayer absents the Al_2_O_3_ layer. Therefore, we designed the thickness to only 5 nm. The layer structure with an alternating low and high refractive index combination can improve reflectivity [25]. The greater the difference between the two refractive indices, the higher the reflectivity of the stack. We chose TiO_2_ as it has the highest refractive index material in the visible and near IR regions. The refractive index of MgF_2_ is almost the lowest. However, MgF_2_ deposited under unheated substrate temperature conditions may absorb water vapor into the film to form MgO [26]. Therefore, we chose SiO_2_ as the low refractive index material. The layer stack materials of amorphous dioxides have less optical absorption [27]. Moreover, the formation of mixed oxide bonds, -Ti-O-Si- [28], at the layer interface can increase the adhesion of the stack.

Wang et al. observed an optical absorption band in the region around 530 nm and speculated the absorption band was caused by silver sulfide nanoparticles [29]. However, Sadovnikov et al. also studied the broad absorption peak at 360–460 nm in the spectrum of silver sulfide nanoparticles. They mentioned that the observed absorption band at ~530 nm was likely due to impurity silver nanoparticles. Moreover, they measured the optical reflectivity of the coarse-crystalline and nanocrystalline silver sulfide powders with average particle sizes of ~500, ~90, and ~60 nm. The bandgaps determined from the optical reflectivity spectra of the Ag_2_S powders were ∼1.6 to 0.6 eV. The bandgap decreases with increasing particle sizes [30]. Figure 7 shows the ~480 nm wavelength at the lower reflectivity of Sample 86 nm is shorter than ~500 nm of Sample 130 nm. That illustrates that the binding energy of Sample 86 nm is larger than that of Sample 130 nm, agreeing with the study of Sadovnikov et al.

When the silver atom loses the electron to become an Ag ion and chemically react with H_2_O, silver oxide, such as Ag_2_O_3_, Ag_3_O_4_, AgO, will be produced. Peter et al. said all silver oxides, AgO_x_ (x > 1), are potent oxidants, yielding dark brown [31]. Moreover, Ahmad et al. observed that the silver oxide nanowire thin films strongly absorb a broad visible band centered at ~530 nm [32]. Therefore, the silver sulfide and silver oxide produced on the silver mirror can decrease the reflectivity by approximately 500 nm. Sample 86 nm was slightly yellowish after sulfurization. Its spectrum has the lower reflectivity in the 400 to 570 nm region and the lowest of 58% at 480 nm shown in Figure 7. When the Ag layer was thicker than 130 nm, the sulfurization process affected the silver layer’s optical quality. Sample 130 nm resulted in a somewhat decreased reflectivity, by approximately 500 nm. It was not easy to distinguish the film quality between the un-sulfurized and sulfurized samples by the camera. However, the spectrometer could detect them, as shown in Figure 7. Furthermore, through the lamp above our laboratory, we could observe that this sample had a small amount of transmitted light, indicating that the reflectivity of the silver films was able to be higher. Hence, an increase in the thickness of the Ag layer was required.

For this, the thickness of the Ag layer was increased to 200 nm to make the sample opaque. Therefore, a spectrometer measured its reflectivity to prove the inference further. Figure 7 shows a highly consistent relative reflectivity between the unsulfurized and sulfurized samples. The average reflectivity was still greater than 97%, though a little reduced at ~500 nm, indicating that the 200-nm thickness of the third layer Ag can be used as a candidate thickness for making telescope mirrors.

When a spectrometer measures a pure Ag-coated sample, the shorter the wavelength, the lower the reflectance when the spectrum is less than 480 nm; additionally, pure silver is sensitive to the sulfurization process. The tarnished black silver is mainly due to the reaction of H_2_S in the air with the Ag coating to form Ag_2_S, which absorbs light in the visible region [23].

From the above results, the enhancement layer, SiO_2_/TiO_2_/SiO_2_/TiO_2_, can improve optical reflectivity and prevent the production of silver sulfide and silver oxide under air circumstances. Sample 200 nm, after 16-month storage, had only some pinholes, and its reflectivity was un-degraded. Additionally, the multilayer deposition of the primary and secondary mirrors followed the process.

**Figure 12:**
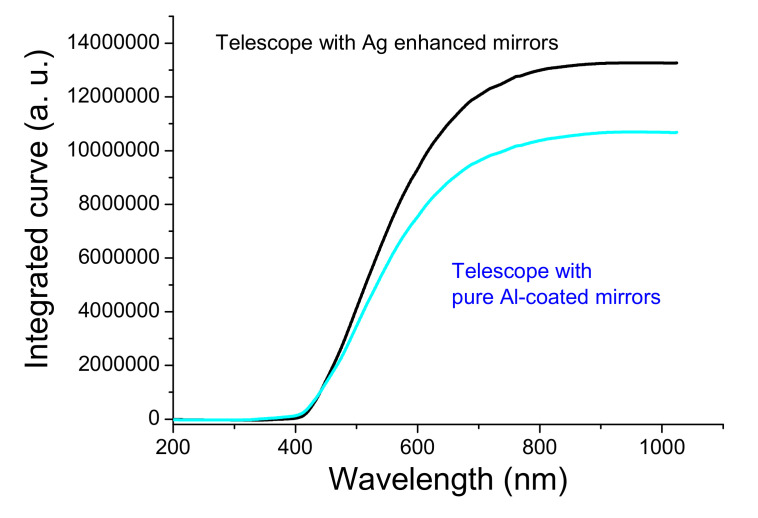
Integrated curves of the spectra of two exit pupils from double telescopes.

The primary and secondary mirrors still kept high reflectivity after six month storage. For comparing the double telescope’s spectra, the prominent white object almost uniformly reflected the visible and near-IR light. However, the outdoor ambient light at five o’clock in the afternoon on a cloudy day illustrated the spectra through the two telescopes, as shown in Figure 11. The maximum intensity was at the 500-nm wavelength. We evaluate the color temperature of ~5796 K using Wien’s displacement law (λ_peak_*T* = 2.898 × 10^−^^3^ mK), where λ_peak_ is the wavelength at the maximum intensity, and *T* is the color temperature. As the spectrum intensity curves are related to the color temperature and some peak absorption by humidity (H_2_O, CO_2_, and O_2_ [33]), we integrated the curves shown in Figure 12. The intensity maxima are 1.328 × 10^7^ and 1.069 × 10^7^ at about 1000-nm wavelength. The ratio of the different integrated intensity to the maxima intensity of 1.328 × 10^7^ is ~19.5%, which approaches those that were calculated by the normalized intensity difference of about 19%. It is less than the theory reduced intensity of 22.6%, as mentioned above, due to the optical scattering and absorption of the enhanced multilayers above the silver layer [34]. The diffuse reflection produced by the optical scattering can seriously reduce the specular reflective image [35]. The excellent performance of the imaging system has low optical scattering. The surface roughness of Ag-enhanced telescope mirror is 1.18 nm, which is better than of the Al-deposited primary mirror of 2.63 nm, the relative total diffuse reflectance is 0.25% to 0.04% for 400-nm to 1000-nm wavelength, when evaluated by (4πσ/λ)^2^, where σ is the surface roughness, and λ is the wavelength [36]. Although the diffuse reflectance is small, the Ag enhancement process can reduce the surface roughness, which may be due to a suitable IAD. Nevertheless, the reflectivity of the Ag-enhanced mirror indeed improved that of Newtonian type telescope by ~20%.

## 5. Conclusions

This study has designed and demonstrated a silver enhanced multilayer for a Newtonian type telescope. Compared to a general telescope made of Al-coated mirrors, this paper shows that the proposed new Ag-coating technology has improved the reflectivity in the 400–500 nm spectral region and increased averaged reflectivity up to 20%. Additionally, when the Ag-coated mirrors were subjected to environmental tests with humidity H = 60–85% and temperature T = 10–35 °C for more than six months, these Ag-enhanced mirrors resisted moisture and inhibited sulfurization without degrading optical performance. Moreover, the thicker silver layer of 200 nm benefitted the reflectivity and sulfurization inhibition. The adhesion testing of the Ag-enhanced multilayer deposited on the B270 glass substrate can pass ASTM 5B degree due to the Al_2_O_3_ buffer layers around the metallic silver coating. These results illustrate that the proposed coating technique can be applied in space, ground, and marine imaging systems.

## Figures and Tables

**Table 1:** Silver-enhanced mirror coating parameters.

Layer	Material	Thickness (nm)	Pressure (Pa)	Deposition Rate (nm/s)	IAD	Temperature (°C)
1	SiO_2_	46.8	1.4 × 10^−2^	0.22	V_b_:119.4 V I_b_:2.76 A	50
2	Al_2_O_3_	20	2.5 × 10^−2^	0.3	V_b_:119.4 V I_b_:3.01 A	50
3	Ag *	86, 130, 200	5.5 × 10^−4^	1	no	60
4	Al_2_O_3_	5	2.5 × 10^−2^	0.16	no	60
5	SiO_2_	54.1	1.4 × 10^−2^	0.22	V_b_:119.4 V I_b_:2.76 A	80
6	TiO_2_	49.5	1.0 × 10^−2^	0.09	V_b_:119.5 V I_b_:2.13 A	105
7	SiO_2_	91.2	1.4 × 10^−2^	0.22	V_b_:119.4 V I_b_:2.76 A	100
8	TiO_2_	12.2	1.0 × 10^−2^	0.9	V_b_:119.5 V I_b_:2.13 A	115

* The third layer’s thickness of Sample 86 nm is 86 nm, Sample 130 nm is 130 nm, and Sample 200 nm is 200 nm, respectively.

**Table 2:** The designed optical admittances, reflectivity, and phase of the silver layers with different thicknesses at the 500-nm wavelength.

Thickness (nm)	Admittance	Reflectivity (%)	Phase
86	0.060-i 2.86	97.43	141.5
130	0.050-i 2.87	97.83	141.6
200	0.05-i 2.87	97.85	141.9

## Data Availability

The data are included in the article.
